# Prevalence characteristic of BVDV in some large scale dairy farms in Western China

**DOI:** 10.3389/fvets.2022.961337

**Published:** 2022-07-28

**Authors:** Kang Zhang, Jingyan Zhang, Zhengying Qiu, Kai Zhang, Fenfen Liang, Qiaoni Zhou, Lei Wang, Jianxi Li

**Affiliations:** ^1^Lanzhou Institute of Husbandry and Pharmaceutical Sciences, Chinese Academy of Agricultural Sciences, Lanzhou, China; ^2^Department of Veterinary Sciences, Gansu Agricultural University, Lanzhou, China; ^3^Shenzhen Bioeasy Biotechnology Co., Ltd., Shenzhen, China

**Keywords:** bovine viral diarrhea virus, bulk tank milk, prevalence characteristic, the large scale dairy farm, Western China

## Abstract

The aim of this study was to analyze the prevalence characteristic of Bovine viral diarrhea virus (BVDV) in some large scale dairy farms in Western China. BVDV was detected in 30 samples of bulk tank milk (BTM) collected from 30 large dairy farms in 7 provinces of western China, 93.33% (28/30) of the farms were infected with BVDV, and S/P ratio was over 0.3 in 28 positive farms. The individual status was further estimated in the dairy farm (No. 10) with the highest positive rate (S/P ratio = 1.37) and the dairy farm (No. 17) with the lowest positive rate (S/P ratio = 0.39). Two hundred cows were, respectively, selected from calf, young cows and lactating cows in farm No. 10 and farm No. 17 and the serum sample of each enrolled cow was collected. The individual positive rate of serum antibody (Ab) was 87.17% (523/600) in farm No. 10 and 31.33% (188/600) in farm No. 17. The individual positive ratio of serum antibody in calves, young cows and lactating cows were 41.75 % (167/400), 58.75% (235/400) and 77.25% (309/400), respectively. BTM Ab of farm No. 10 has an S/P ratio more than 1.0, which indicated there were emergent or persistent infection (PI) cases, and further test showed that PI cases were 0.51% in farm No. 10. Pathogens were positive in 42.34% (163/385) of nasal mucus samples collected from cows with respiratory symptom, and BVDV cases were 57 in 163 positive samples. Three strains of NCP BVDV-1, one strain of CP BVDV-1, one strain of NCP BVDV-2 and one strain of CP BVDV-2 were successfully isolated. Phylogenetic analysis revealed that the subtypes of BVDV currently prevalent in western China were BVDV-1a, BVDV-1m, BVDV-1q and BVDV-2. The findings suggested that the BVDV infection is serious in some Large Scale Dairy Farms in Western China.

## Introduction

Bovine viral diarrhea virus (BVDV) mainly infects animals such as cattle, sheep and pigs (1). At present, BVDV is widely distributed over the world, including many developed countries with a cattle industry. For example, BVDV is endemic in Australia with annual economic losses up to 1.14 billion Australian dollars (2).

BVDV is composed of enveloped nucleocapsid and nucleic acid, which is a representative species of the genus pestivirus in Flaviviridae family (3). BVDV is divided into cytopathic (CP) and non-cytopathic (NCP) according to whether the virus is pathogenic to cultured cells (4). Among them, CP BVDV often results in cell apoptosis (5), however, NCP BVDV is capable of establishing persistent infections (6). According to the difference in the 5′-UTR gene sequence, BVDV can be divided into two genotypes, namely BVDV-1 and BVDV-2 (7). Twenty-one subtypes of BVDV-1 (1a-1u) and 4 subtypes of BVDV-2 (2a-2d) have been identified (8, 9). Another new BVDV genotype has been identified with the serum of diseased cattle in Europe, namely HoBi-like BVDV-3, which consists of two sub-groups of Thai origin and Brazilian origin (10).

BVDV was first identified and isolated in New York State in 1946 (11). Since then, BVDV has been found in various parts of the world. Currently, the infection rates are particularly serious in a lot of countries, and the BVDV seroprevalence has reached over 50% (12). BVDV seroprevalence was 75–84% in North American countries (13), 23–52% in Middle East countries (14, 15), 22.2–90% in South American countries (16, 17), 47.4–53.27% in European countries (18, 19), 51–80.7% in Asian countries (20, 21), and more than 89% in Australian countries (22, 23). The detection of BTM BVDV Ab can also determine the overall antibody status of the dairy herd, which can replace the detection method of serological samples and reduce the cost of epidemiological investigation (24). For example, the positive ratio of BTM BVDV antibody in Ireland is as high as 73% (25). The results of molecular epidemiological investigation in recent years have also confirmed the widespread prevalence of BVDV in multi-genotype, which brings difficulties to the current prevention and control of BVDV. The prevalence of subtypes varies from region to region. In the United States, three main subtypes (BVDV-1a, 1b, and BVDV-2a) have been identified. PI animals are mainly persistently infected with BVDV-1b (26). In Poland, two genotypes, BVDV-1b and 1d, were mainly prevalent (27). Turkey and Argentina have the same epidemic subtypes, mainly BVDV-1a, 1b and BVDV-2 (28, 29). The genotypes BVDV-1b, BVDV-1m and BVDV-1q were the most prevalent in China (30). However, most of the previous studies involved beef cattle and yaks in western China. There are no systematic reports on the prevalence of BVDV in large scale dairy farms in western China.

This study analyzed the prevalence characteristic of BVDV in some large scale dairy cows in 7 provinces of western China for the first time, including Gansu, Ningxia, Xinjiang, Inner Mongolia, Shaanxi, Tibet and Qinghai. The respiratory pathogens were detected and the incidence of BVDV infection was calculated. 93.33% of the farms were infected with BVDV in 30 dairy farms, and the individual positive rate of serum Ab was 87.17% in the farm with the highest positive ratio, and 31.33% in the farm the lowest positive ratio. The subtypes of BVDV currently prevalent in western China were BVDV-1a, BVDV-1m, BVDV-1q, and BVDV-2. The findings suggested that the BVDV infection is serious in some Large Scale Dairy Farms in Western China, and provide some evidences for clarifying the genetic evolution relationship between different strains, trace the source of virus transmission and predict the epidemic trend of the virus, so as to accumulate experience in the development of BVDV vaccines.

## Materials and methods

### Study area and animals

**Figure 1:**
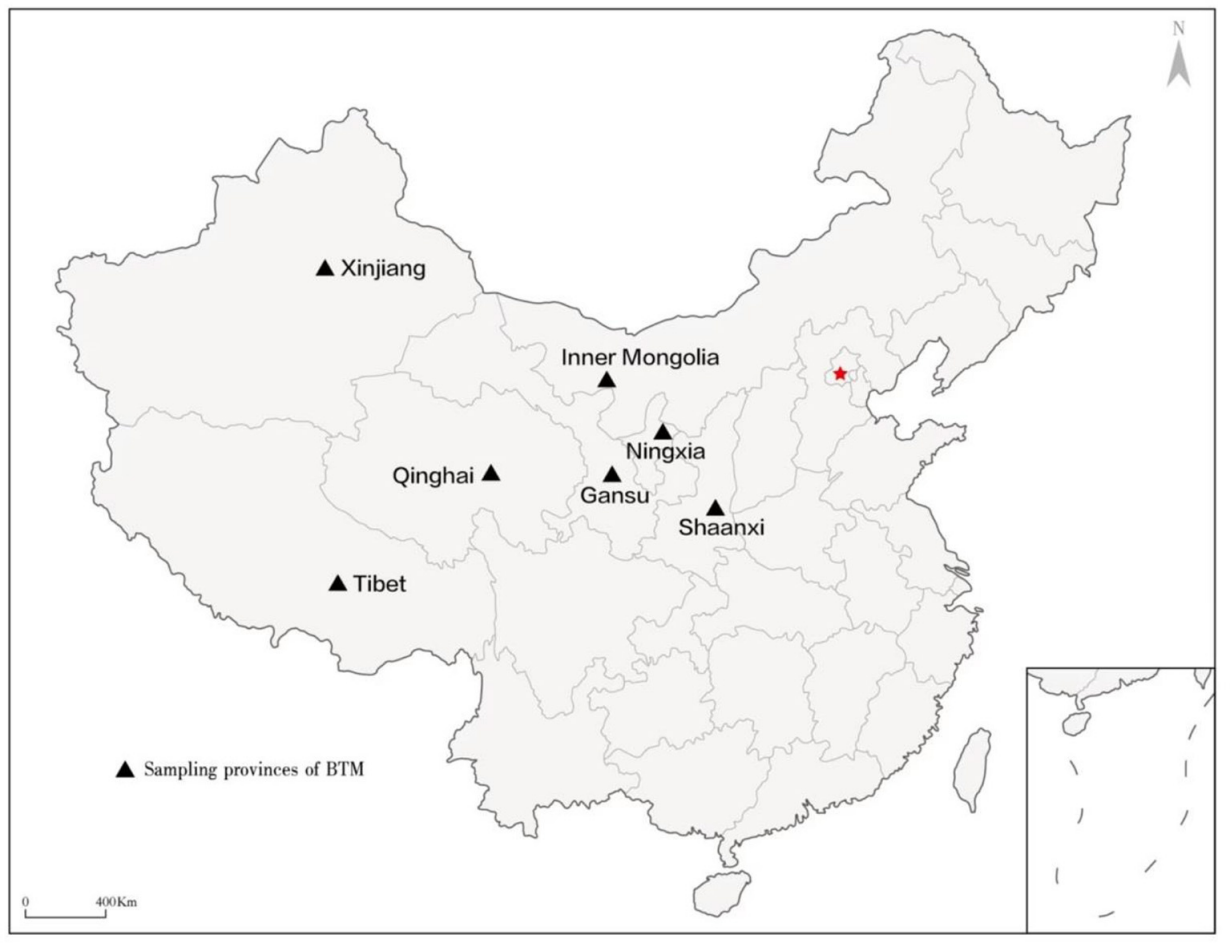
Sampling regions (provinces in western China) involved in this study.

The western region is the main milk producing area in China. In order to get a clearer understanding of the prevalence characteristic of BVDV in some large scale dairy farms in western China. In the test on the herd status of BVDV positive, 30 dairy farms with a size of over 1,000 dairy cows were enrolled in the study, and they located in 7 provinces including Gansu, Ningxia, Xinjiang, Qinghai, Shaanxi, Inner Mongolia and Tibet in western China between November 2019 and February 2022 (Figure 1). In the test on the individual status of BVDV positive, claves <3-month-old were not enrolled because the maternal antibody can interfere with the real result of self-antibody. Two hundred cows were randomly enrolled in per age group, including three age groups (calves between 4 and 6 months old, young cows between 6 and 18 months old, and lactating cows more than 28 months old) in the dairy farm with the highest positive ratio and in the dairy farm with the lowest positive ratio, respectively. Cows in these 30 dairy farms were not injected with BVDV vaccine, while other vaccines (such as foot-and-mouth disease vaccine) were given according to normal immunization procedures. All of experiments on animals were conducted under the permission of the Animal Ethics Committee of Lanzhou Institute of Husbandry and Pharmaceutical Sciences of CAAS (the permission number: SYXK (Gan) 2019-0002).

### Samples collection and BVDV antibody detection

Thirty BTM samples were enrolled for detection of the BVDV-status of each farm. After the BTM samples were transferred to the Lab., they were centrifuged at 2,000 × g for 15 min, three different layers appeared in the tubes, the middle layer was collected and stored in a sterile eppendorf tube at −20°C for detection of BVDV Ab with the BVDV Antibody Detection Kit (IDEXX, USA). In brief, after the milk sample was taken out from the coated plate, set negative control (2 repeats) and positive control (2 repeats), added 25 μL negative standard or positive standard in the corresponding wells, then the 100 μL diluent was added. In test wells, 100 μL processing milk samples were added. After finishing the above operations, the plates were incubated at 21°C for 90 min. The liquid in wells were sucked out, and wells were washed 5 times with a washing solution. After 100 μL of enzyme marker were added, plates were incubated at 21°C for 30 min, then discarded the liquid, and washed for 5 times. Substrate of 100 μL was added in each well, shaken and mixed. After reaction 15 min at 21°C in dark, stop solution of 100 μL were added in each well, and the optical density was read with a microplate reader (GENE, USA). The test results was valid when the value of PCx minus NC $\bar{\text{x}}$ was over 0.15, PC $\bar{\text{x}}$ is the average value of positive samples OD450 nm, and NC $\bar{\text{x}}$ is the average value of negative samples OD450nm, furthermore, NC $\bar{\text{x}}$ ≤ 0.25. The positive samples were determined using S/P ratio which is calculated using the following equation: S/P = (Sample OD450 nm-NC $\bar{\text{x}}$)/(PC $\bar{\text{x}}$-NC $\bar{\text{x}}$) × 100%, and S/P ≤ 0.20 indicates negative, 0.20 ≤ S/P < 0.30 indicates suspect, S/P ≥ 0.30 indicates positive.

Subsequently, the individual status of BVDV positive cows were investigated in the farm with the highest positive ratio of BTM BVDV Ab, and in the farm with the lowest positive ratio of BTM BVDV Ab. A total of 1,200 blood samples were collected and 200 blood samples in each age group per enrolled dairy farm. The blood samples were centrifuged at 3,500 × g for 15 min to obtain serum, and stored at −20°C for further analysis. The detection of BVDV Ab was examined with the BVDV Antibody Detection Kit (IDEXX. USA). The results was determined as same as the test of BTM BVDV Ab, the test procedure was only a little difference from the test of BTM BVDV Ab, the serum sample of 25 μL needed to dilute with dilution regent with a ratio of 1:4. Meanwhile, 2,140 blood samples were collected from the farm with the highest positive ratio of BTM BVDV Ab.

Three hundred and eight-five nasal mucus were collected from the clinical cases based on clinical symptoms of depression, cough, short rapid breathing, excessive lacrimation, excessive nasal secretion, and watery diarrhea in 30 dairy farms (Table 1). After being transferring to Lab., samples were stored at −80°C for RT-PCR/PCR and virus isolation.

**Table 1:** The collection of nasal mucus.

**No**.	**Samples**	**Number**	**Origin**	**Sampling time**
1	Nasal mucus	24	Gansu	2019
2	Nasal mucus	45	Gansu	2020
3	Nasal mucus	31	Gansu	2021
4	Nasal mucus	28	Shaanxi	2020
5	Nasal mucus	34	Shaanxi	2021
6	Nasal mucus	22	Xinjiang	2019
7	Nasal mucus	20	Xinjiang	2020
8	Nasal mucus	18	Xinjiang	2021
9	Nasal mucus	37	Ningxia	2020
10	Nasal mucus	34	Ningxia	2021
11	Nasal mucus	16	Inner Mongolia	2021
12	Nasal mucus	14	Qinghai	2020
13	Nasal mucus	25	Qinghai	2021
14	Nasal mucus	17	Tibet	2019
15	Nasal mucus	8	Tibet	2020
16	Nasal mucus	12	Tibet	2021

### RT-PCR and PCR

According to the RNA/DNA extraction kit instructions, DNA/RNA was extracted from fresh blood and nasal mucus of cattle. Bacterial DNA was extracted with the Bacterial DNA Kit (Omega Bio-Tek, USA) according to the manufacturer's recommendations. Under the instructions, the blood samples pooled in groups of 8 cows were used for molecular detection of BVDV with Real PCR^*^BVDV RNA Test kit (IDEXX, USA). This method is suitable for screening BVDV positive dairy animals. To evaluate whether BVDV-positive cattle are PI animals, blood samples were collected and repeated detection by RT-PCR again four weeks later. It was considered to be PI cattle if the BVDV gene is positive. Pathogens such as BVDV, Bovine parainfluenza virus type3 (BPIV3), Bovine respiratory syncytial virus (BRSV), Bovine coronavirus (BCoV), *Pasteurella multocida* (*Pm), Mannheimia haemolytica* (*Mh*), and *Mycoplasma bovis* (*M. bovis*) were detected in samples from clinical manifestations of the respiratory tract with the multiplex TaqMan real-time fluorescent quantitative PCR kit (Bioeasy, China). The PCR primer pairs were used for detection of Infectious bovine rhinotracheitis virus (IBRV) and the amplification of the 5′-UTR fragment of BVDV of each isolated virus (31).

### Cell culture and virus isolation

An appropriate amount of PBS buffer was added to the nasal mucus and whole blood samples that are identified as BVDV-positive. After fully dissolving and suspending, the supernatant was collected by low-speed centrifugation. The monolayer of MDBK cells was adsorbed at 37°C for 2 h and then supplemented with DMEM maintenance solution containing 4% fetal bovine serum, cultured at 37°C and 5% CO_2_ for 3 days, and the cytopathic condition was observed every day. At the same time, a negative control group was set up, that is MDBK cells without sample treatment solution inoculation. Continuous blind transmission to 12 generations, extracted viral RNA, and used RT-PCR technology to amplify the BVDV 5′-UTR fragment. The RT-PCR product was detected by 1.0% agarose gel electrophoresis and recovered from the gel, and then cloned into the pMD18-T vector. The recombinant plasmid identified as positive was sent to Sheng gong Bioengineering (Shanghai) Co., Ltd. for sequencing.

### Immunofluorescence detection

The 12-passage blind virus was inoculated into a 24-well plate containing monolayer MDBK cells, and the uninoculated normal cells were used as negative control. Twenty-four-well plates were placed in a 5% CO_2_ incubator at 37°C for 12 h, then the cells were fixed with 4% paraformaldehyde after the nutrient solution was discarded and washed with PBS 3 times. After washing 3 times with PBS, cells in the experimental group and control group were stained according to BVDV FITC (VMRD, CJ-F-BVD-10 mL) method combined with fluorescent antibody staining. After washing with PBS 3 times, the nuclei were stained with DAPI. After washing with PBS 3 times, cell staining was observed under a fluorescence microscope with a 24-well plate.

### Phylogenetic analysis

The 5′-UTR regions of the isolated virus were amplified and sequenced. Phylogenetic reconstructions for genetic typing of the viral samples were compiled using a 289 nucleotide region of the 5′-UTR. Multiple sequences were aligned using the Clustal V program with the corresponding regions of BVDV-1, BVDV-2, BVDV-3, Border Disease Virus (BDV) and Classical Swine Fever Virus (CSFV) reference sequences retrieved from GenBank, and a total of 43 gene sequences be gained (including 2 gene sequences of CSFV, 1 gene sequence of BDV, and 40 gene sequences of BVDV). Phylogenetic and molecular evolutionary analyses were conducted using MEGA version 7.0 package (DNAStar Inc., USA). Nucleotide sequences were aligned using the Clustal V method, and phylogenetic trees were constructed.

### Statistical analysis

All data were managed in Microsoft Excel. The Chi-square test was adopted to test the significance of overall seroprevalence and individual differences, and *p* < 0.05 as the lowest level for statistical significance.

## Results

### Seroprevalence of BVDV

Thirty dairy farms were enrolled from 7 provinces to investigate the prevalence characteristics of BVDV in dairy cows (Gansu, Ningxia, Xinjiang, Qinghai, Shaanxi, Inner Mongolia and Tibet) in western China. Firstly, 30 dairy farms' BTM samples were analyzed with a BVDV Ab detection kit. The result of ELISA showed that 93.33% of the BTM samples (28/30) were positive (Table 2).

**Table 2:** Detection results of BTM BVDV antibody by ELISA in western China.

**Farm no.**	**Location**	**Farm size**	**S/P**	**Status**
1	Gansu	3,562	0.79	P
2	Gansu	3,015	1.12	P
3	Gansu	2,223	0.75	P
4	Gansu	3,812	0.79	P
5	Gansu	3,056	0.78	P
6	Gansu	2,081	0.42	P
7	Gansu	4,580	0.65	P
8	Gansu	1,765	1.29	P
9	Gansu	1,437	0.47	P
10	Ningxia	2,140	1.37	P
11	Ningxia	4,125	0.69	P
12	Ningxia	1,756	0.46	P
13	Xinjiang	4,014	0.87	P
14	Gansu	2,824	1.17	P
15	Ningxia	1,890	1.03	P
16	Shaanxi	2,518	0.93	P
17	Qinghai	1,781	0.39	P
18	Inner Mongolia	1,654	0.59	P
19	Xinjiang	3,218	0.52	P
20	Xinjiang	1,782	0.11	N
21	Xinjiang	2,145	1.07	P
22	Xinjiang	2,622	0.42	P
23	Shaanxi	2,247	0.56	P
24	Tibet	1,578	1.07	P
25	Gansu	3,851	0.21	N
26	Xinjiang	3,817	0.51	P
27	Xinjiang	3,197	0.52	P
28	Xinjiang	2,704	0.62	P
29	Inner Mongolia	2,178	0.42	P
30	Ningxia	1,462	0.67	P

Interpreting S/P Ratios, S/P > 1.0 indicates active or recent PI exposure; S/P > 0.75 indicates relatively recent herd BVDV exposure; S/P > 0.25 indicates some random or historic BVDV exposure; S/P < 0.25 indicates naive herd. Swan Veterinary Services provides reference for antibody testing in Bulk Milk Tank Testing.

**Figure 2:**
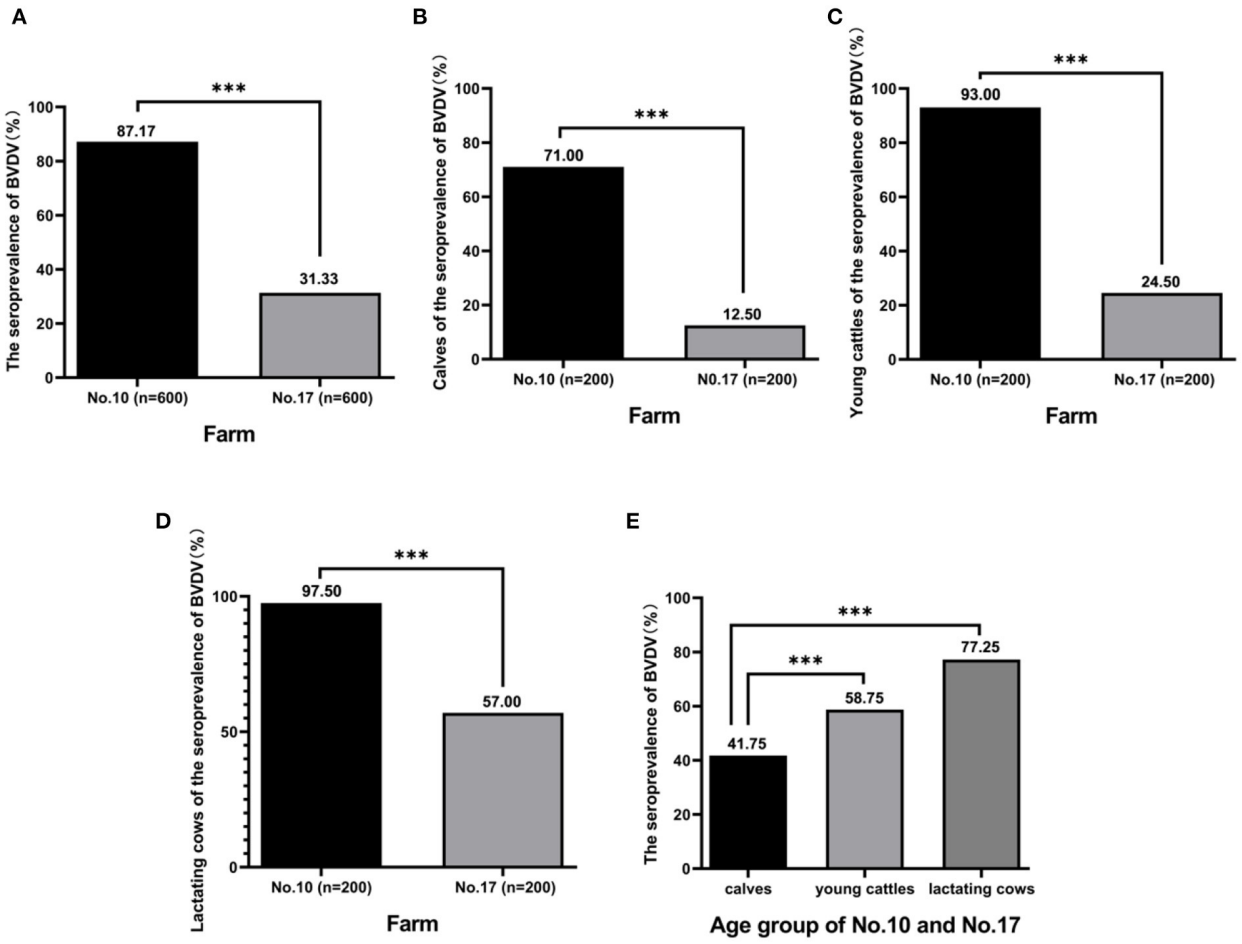
The seroprevalence of BVDV in individual animals. A total of 1,200 blood samples were collected from farm No. 10 and farm No. 17 including calves, young cattle, and lactating cows, and antibody against BVDV was detected by BVDV Antibody Detection Kit. The seroprevalence of BVDV in two farms was analyzed **(A)**, rate of calves was analyzed **(B)**, rate of young cattle was analyzed **(C)**, rate of lactating cows was analyzed **(D)**, and rate of calves, young cattle, and lactating cows was analyzed, respectively **(E)**. ***Indicates *P* < 0.001.

It is generally accepted that BTM antibody levels are positively correlated with the within-herd BVDV seroprevalence of lactating cows. The more animals contributing to the sample with antibodies, the higher the antibody S/P ratio will be. Notice that the S/P ratio is higher from milk samples where in a high proportion of animals is seropositive with the BVDV antibody detection kit. Therefore, the next step was to investigate the seroprevalence rate of BVDV in the individual status of the farm No. 10 with the highest positive rate (S/P ratio = 1.37) and the farm No. 17 with the the lowest positive rate (S/P ratio = 0.39). Blood samples were collected from 2 farms for 3 age groups, 200 blood samples were collected from each age group, 1,200 blood samples were collected in total. The results showed that 87.17 % (523/600) of animals in farm No. 10 and 31.33 % (188/600) of animals in farm No. 17 were positive (Figure 2A), and the positive rate of farm No. 10 was significantly higher than that the positive rate of farm No. 17 (*p* < 0.05). The positive rate of BVDV infection of calves in two farms was, respectively, 71.00% (142/200) and 12.50% (25/200) (Figure 2B), and farm No. 10 positive rate was significantly higher than the other (*p* < 0.05). The positive rate of BVDV infection in young cattle of the herds was, respectively, 93.00% (186/200) and 24.50% (49/200) (Figure 2C), and farm No. 10 positive rate was significantly higher than the other (*p* < 0.05). The positive rate of BVDV infection of lactating cows of these herds was, respectively, 97.50% (195/200) and 57.00% (114/200) (Figure 2D), and farm No. 10 positive rate was significantly higher than of the other (*p* < 0.05). Furthermore, the results of comparing the positive rates for each age group of the two farms was found that the positive rate of BVDV infection of calves, young cattle, and lactating cows was 41.75 % (167/400), 58.75% (235/400), and 77.25% (309/400), and the rate of young cattle and lactating cows was significantly higher than that of calves (*p* < 0.05) (Figure 2E).

### Prevalence of BVDV PI animals

Since the BTM antibody of farm No. 10 has an S/P ratio more than 1.0, it indicated active or recent exposure to BVDV. Therefore, 2,140 blood samples were collected from farm No. 10, and PI animals were screened with Real PCR^*^BVDV RNA Test kit (IDEXX, USA). One hundred and fifty six positive animals were found with an infection rate of 7.29%. Four weeks later, blood samples from 106 positive animals were collected again. Repeated detection results showed that 11 cows were considered as PI animals, with a PI rate of 0.51%.

### Detection of respiratory pathogen

The nasal mucus of 385 cows with respiratory symptoms was collected and the pathogen was detected by PCR or RT-PCR. Pathogens were positive in 42.34% (163/385) of the collected nasal mucus samples, and BVDV cases were 57 in 163 positive samples. The isolated infection rate of BVDV was 4.15% (16/385), while the rate of BVDV infection combined with IBRV, *M. bovis, Pm, Mh*, BCoV/*Mh, Pm*/*Mh*, or BCoV/BPIV3 BRSV was 5.19% (20/385), 2.08% (8/385), 1.04% (4/385), 0.78% (3/385), 0.26% (1/385), 0.78% (3/385), and 0.52% (2/385), respectively. Therefore, BVDV is one of the most important pathogens, and co-infections with other pathogens were common. Besides, the detection rate of *M. bovis* and *Pm* was also very high.

### Cultivation and identification of isolated strains

**Figure 3:**
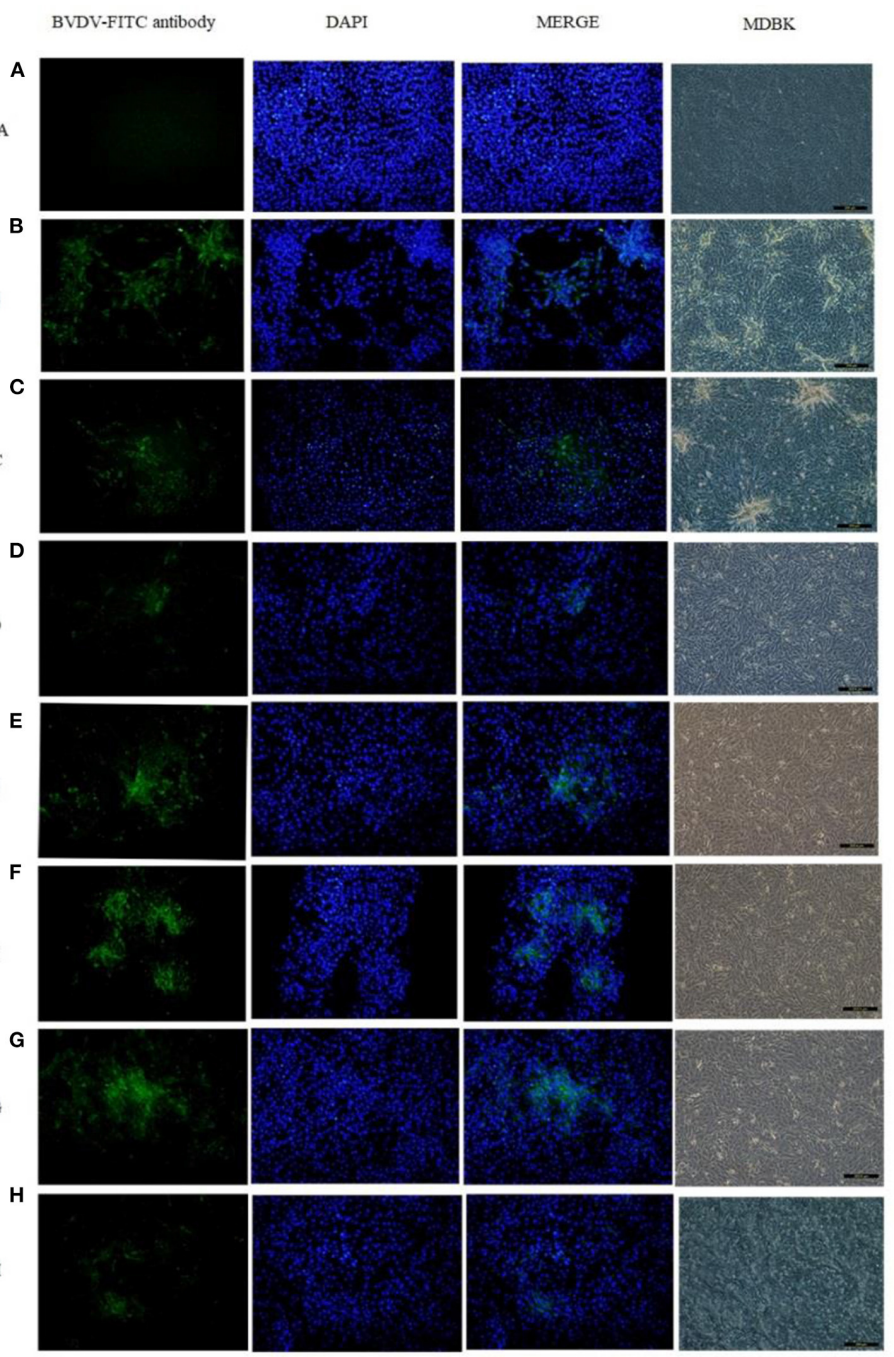
Total six strains were isolated, only GSLY and NXWZ isolates generated cytopathic effects, the others had no cytopathic effect on cells. All separated cultures were observed under the fluorescence microscope, and green fluorescence appeared in the cytoplasm, **(A)**: the MDBK cell as negative control, **(B)**: the cytopathic of NADL strain as positive control, **(C)**: the cytopathic of GSLY strain, **(D)**: the non-cytopathic of GSLZ strain, **(E)**: the non-cytopathic of SXHY strain, **(F)**: the non-cytopathic of XJFK strain, **(G)**: the non-cytopathic of NXYC strain, **(H)**: the cytopathic of NXWZ strain.

**Figure 4:**
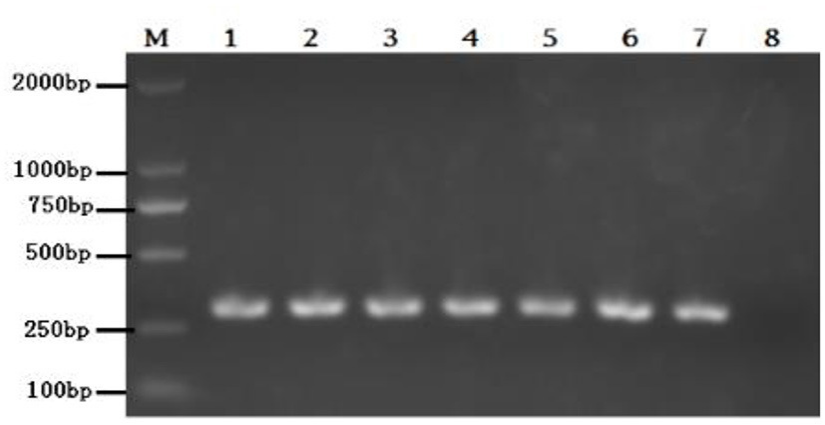
Lane M: weight size marker (2,000, 1,000, 750, 500, 250, 100 bp), lanes 1–6: isolate GSLY, GSLZ, SXHY, XJFK, NXYC, and NXWZ, respectively, lane 7: positive control; lane 8: negative control.

Eighteen BVDV positive samples (including 1 whole blood and 17 nasal mucus) were cultured, and BVDV was isolated and identified after 12 passages on MDBK cells (Figure 3). The isolates were identified by RT-PCR (Figure 4), and then sequenced and analyzed. Six BVDV strains were isolated, and named as GSLY, GSLZ, SXHY, XJFK, NXYC, and NXWZ, respectively. Only GSLY and NXWZ had cytopathic effects among cells. The rest did not generate obvious cell lesion. Details of clinical symptoms were shown in Table 3. In the fluorescence immunoassay, the virus-inoculated cells showed specific green fluorescence under the fluorescence microscope, and green fluorescence mainly appeared in the cytoplasm, while normal cells that had not been inoculated with BVDV showed no fluorescence.

**Table 3:** Status of BVDV biotypes and genotypes in western China.

**Name**	**Samples**	**Origin**	**Clinical symptoms**	**Biotype**	**Genotype**
GSLY	Nasal mucus	Gansu	Pneumonia	CP	1a
GSLZ	Nasal mucus	Gansu	Pneumonia	NCP	2
SXHY	Nasal mucus	Shaanxi	Pneumonia	NCP	1q
XJFK	Nasal mucus	Xinjiang	Pneumonia	NCP	1m
NXYC	Nasal mucus	Ningxia	Pneumonia	NCP	1q
NXWZ	Blood of PI animal	Ningxia	None	CP	2

### Genetic analysis of isolated viruses

**Figure 5:**
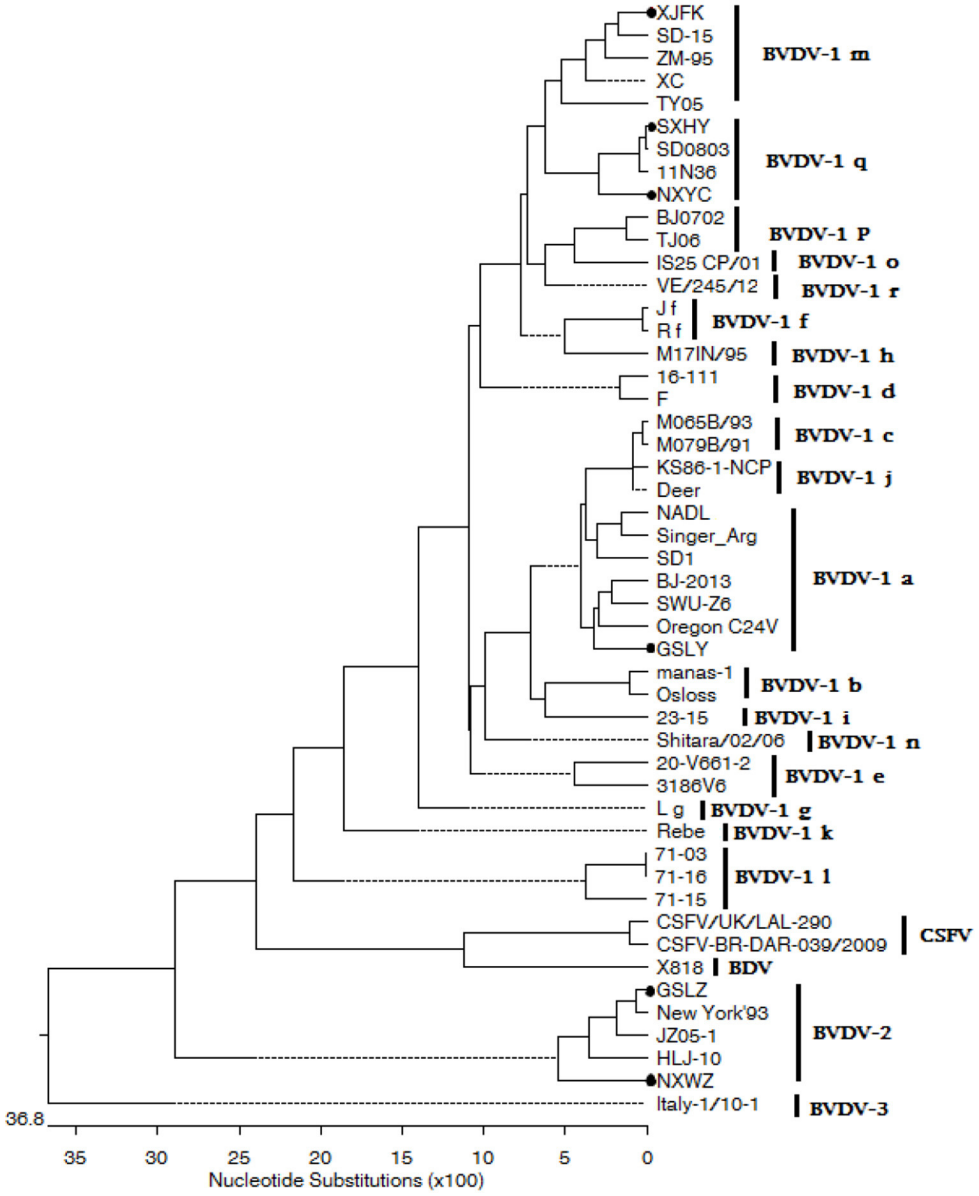
The phylogenetic tree based on 5′-UTR of BVDV strains. The evolutionary history was inferred using the neighbor-joining method. The sequences of GSLY, GSLZ, SXHY, XJFK, NXYC, and NXWZ isolates were determined in this study; sequences of other strains were obtained from GenBank.

The 289 bp fragment of the 5′-UTR regions of each isolated virus was amplified and sequenced. Evolutionary analyses conducted in MEGA version 7.0 based on the sequence in the 5′-UTR showed that four BVDV strains were BVDV-1, and the genotypes were BVDV-1a, BVDV-1m, and BVDV-1q. Furthermore, two isolates BVDV-2 strain were isolated and identified (Figure 5).

## Discussion

Bovine viral diarrhea (BVD) is one of the serious diseases endangering the cattle industry in the world (32, 33). It is listed as a statutory notifiable animal disease by the International Epizootic Office (OIE) and a ClassIII animal disease in China. Since 1980, when BVDV was first isolated from China, a large number of epidemiological investigations showed that BVDV infection has been reported in most areas of China (30). Although western China is rich in forage and land resources and has the advantage of developing the dairy cattle industry, there are few epidemiologic investigations on BVDV. Therefore, systematic epidemiological investigation of BVDV in western China was carried out. The genotype of the virus was identified through genetic evolution analysis, and the biological type of the virus was isolated and identified, providing theoretical basis and material basis for the prevention and control of BVDV in western China.

Currently, antibody detection methods for milk samples and subsequent detection methods for different infectious diseases have been developed. Among them, a highly sensitive and effective method can be used to detect mixed samples, such as BTM (34). The detection of BTM is usually used for establishing bovine health system and evaluating diseases such as BVDV by foreign diagnostic laboratories (24). A lot of practice has proved that the detection of antibodies in BTM plays a key role in the prevention and control of animal diseases (35). In this study, BTM samples from 30 non-BVDV vaccinated farms of dairy cattle that are widely distributed among western China was submitted to prevalence investigation of BVDV. In this representative group of BTM samples, 93.33% of farms were infected with BVDV (Table 2), suggesting that BVDV may be widespread in western China and higher than the national average BVDV antibody of 53% (36). Individual status of the two farms (Figure 2) indicated that there may be widespread infection in farm No. 10, and the positive ratio of BVDV infection in both young and lactating cattle were significantly higher than that in calves. In addition, the positive ratio was higher in lactating cattle than in young cattle, which had a higher prevalence due to the greater lifetime exposure of older cattle to the virus (37, 38). Most importantly, a sudden increase in pneumonia and diarrhea during calves in farm No. 10 has been reported by veterinarians, which may be related to PI cattle.

PI refers to a pathogen that remains in the host for a long time without being cleared. The incubation period is months or years, and some viruses can be carried for life (39). Its symptoms are not clinically evident, and the pathogen may not continuously proliferate, which is a key infection source in maintaining BVDV in dairy farms (40). The results showed that the positive ratio of PI animals in 2,140 from cattle farm No. 10 was 0.51%, which was higher than the PI positive rate (below 0.2%) reported Switzerland (41). The results also verified the existence of PI cattle when the S/P value of BVDV antibody of BTM is >1, indicating that cattle in western China was at high risk of BVDV infection. RT-PCR was found out as an accurate and cost-effective additional method for identifying PI animals in lactating cattle of any age (42). As more emphasis is placed on the identification of potential sources of contamination in dairy farms, this method will also be adopted by more cattle farms to remove PI cattle in certain areas (43).

**Figure 6:**
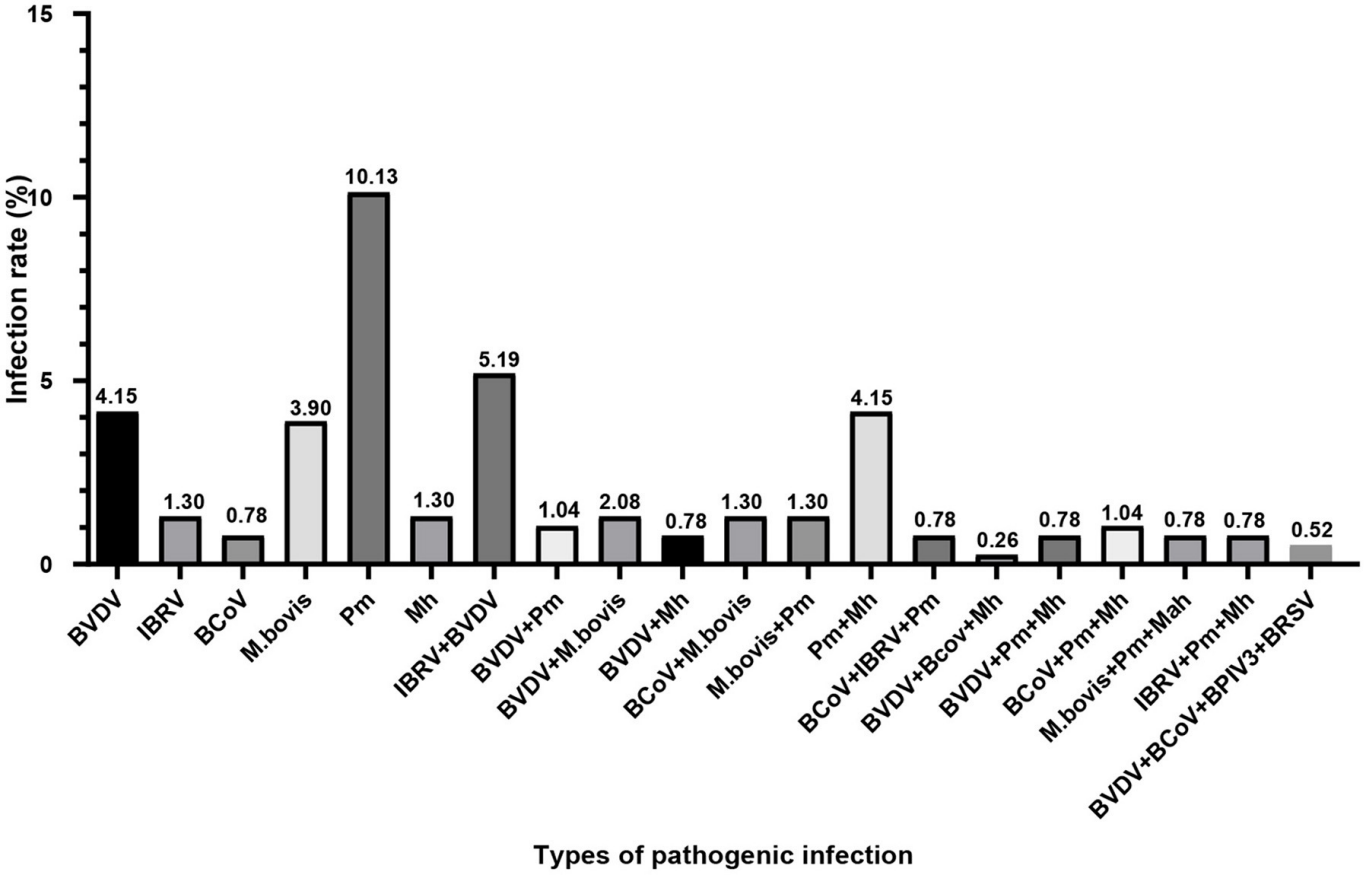
Pathogens of respiratory diseases in nasal mucus samples from all clinical cattle were detected by PCR or RT-PCR. The ratio of each or combined pathogens was analyzed using Chi-square test.

To further investigate the positive infection of BVDV in cattle farms, 385 nasal mucus samples were collected from 30 farms for analysis. The results showed that BVDV was the main pathogen with a high proportion of mixed infection with other pathogens (Figure 6). BVDV can induce immunosuppression, and infected animals tend to promote secondary infections with other pathogens (44). Furthermore, *Mycoplasma* and *Pasteurella multocida* also showed a high positive ratio, which usually causes high mortality in calves with mixed infection and should be of concern to cattle farm (24).

Three strains of NCP BVDV-1, one strain of CP BVDV-1, one strain of NCP BVDV-2 and one strain of CP BVDV-2 were successfully isolated in this study. The results suggested that most clinical isolates of BVDV in China were NCP (45). In natural infection, NCP is more common than CP, and only NCP can cause PI. These two BVDV biotypes play an important role in the pathogenesis of mucosal diseases (46). BVDV is a single-stranded RNA virus with high mutation rate, and its genotypes vary among countries and regions. BVDV-1a, BVDV-1b, and BVDV-2a are the most prevalent in the United States, whereas the 1a, 1b, 1d, 1e, and 1f are widespread in European countries (47–49). BVDV-1b is mainly prevalent in Japan and Korea, while BVDV-1c is mainly predominant in Australia (50–52). Multiple subtypes of BVDV-1 and BVDV-2 have been found in China, with diverse distribution, large geographical area and complex epidemic situation. Previous studies have confirmed that BVDV-1 subtypes 1a, 1b, 1c, 1d, 1m, 1o, 1p, 1q, and 1u, were circulating in susceptible animals in China (30, 53). In this study, BVDV-1a, BVDV-1m, BVDV-1q, and BVDV-2 were also found in western China based on a highly conserved 5′-UTR sequence (Figure 5). Obvious clinical symptoms of respiratory tract were observed in the infected cattle in this cattle farm, which, combined with the respiratory pathogen detection results, could easily be speculated to be related to secondary infections with other pathogens. This is the first time that BVDV-2 genotype strain was isolated from large scale dairy farms in western China. Although BVDV-1 is the dominant genotype in western China, cattle farms are still threatened by the BVDV-2 genotype. Because of the genetic diversity of virus isolates, a vaccine effective in one region may not able to protect against viral infections caused by different virus strains in another region. It is suggested that cattle farms are being immunized with domestic BVDV vaccine should be considered for BVDV control in combination with PI cattle PCR screening strategy.

## Conclusions

The serological investigations showed that the antibody positive ratio of dairy cows in western China was higher than the national average. BVDV was widespread in herds, and NCP was the dominant biotype. In addition, the BVDV-2 genotype was isolated for the first time in large scale dairy farms in western China. Although the BVDV vaccines independently developed in China have been on the market, they all belong to the BVDV-1 genotype. However, the BVDV-2 genotype strains were detected from nasal mucus of infected animal and blood of PI animal this time (Table 3). Therefore, it is suggested to develop the combined vaccine of the BVDV-1 and BVDV-2 genotype for immunization prevention and control in China. This study laid a foundation for further study on vaccination and control strategies of BVDV in western China. Besides, further PI animal screening on larger scale dairy farms in the western China needs to be carried out to determine whether the positive rate is high and evaluate the risk of BVDV infection.

## Data availability statement

The original contributions presented in the study are included in the article/Supplementary material, further inquiries can be directed to the corresponding author/s.

## Ethics statement

All procedures were carried out following guidelines by the Animal Care and Use Committee, Lanzhou Institute of Husbandry and Pharmaceutical Sciences of the Chinese Academy of Agricultural Sciences (the permission number: SYXK(Gan) 2019-0002).

## Author contributions

KZ, JL, JZ, and ZQ designed the study and wrote the manuscript. KZ and QZ participated in sample collection. FL and LW performed the experiments, data analysis, and drafted the manuscript. All authors read and approved the final manuscript.

## Funding

This work was financially supported by the earmarked fund for CARS36, Gansu Province Science and Technology Foundation for Youths (No. 21JR7RA030), the Science and Technology Innovation Project of CAAS Collaborative Innovation (No. CAAS-XTCX2016011-01-09), and Innovation Project of Traditional Chinese Veterinary Medicine and Clinical Science (No. CAAS -ASTIP-2015-LIHPS).

## Conflict of interest

Author QZ was employed by Shenzhen Bioeasy Biotechnology Co., Ltd. The remaining authors declare that the research was conducted in the absence of any commercial or financial relationships that could be construed as a potential conflict of interest.

## Publisher's note

All claims expressed in this article are solely those of the authors and do not necessarily represent those of their affiliated organizations, or those of the publisher, the editors and the reviewers. Any product that may be evaluated in this article, or claim that may be made by its manufacturer, is not guaranteed or endorsed by the publisher.
