# Supplementary material for: Prevalence characteristic of BVDV in some large scale dairy farms in Western China
Source: Front Vet Sci. 2022 Jul 28;9:961337. doi: 10.3389/fvets.2022.961337 (PMC9366859; doi:10.3389/fvets.2022.961337)
Supplement: Supplementary file 1 [file Data_Sheet_1.PDF]

Bulk Milk Tank Antibody Testing

This powerful new assay is designed to provide producers and veterinarians with a simple "average" BVDV antibody level assessment for a population of lactating cows.

The BMT antibody level is directly correlated with individual animal antibody levels. Quite simply, the more animals contributing to the sample with antibodies, the higher the antibody S/P ratio will be. This graph depicts some of the work Hinrich Voges of the Livestock Improvement Coporation performed to validate the assay. Notice that the S/P ratio is higher from dairies wherein a high proportion of animals are seropostive. Further, note that dairies, confirmed to have a lactating PI, all had S/P ratios over 1.0.

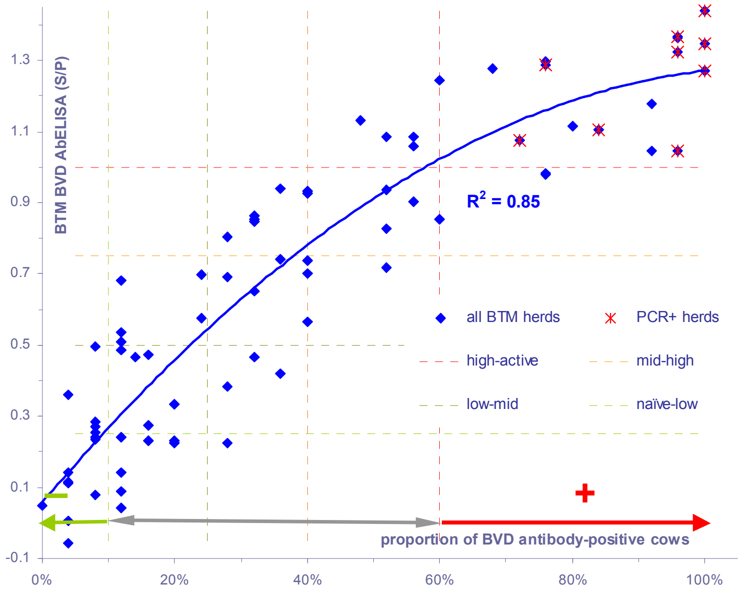

The BMT antibody level in a dairy herd will not fluctuate unless either a PI has recently exposed the herd or there has been an addition of a group of either strongly antibody free or antibody positive animals between sampling events. Day to day variations in the number of animals included within the Bulk Milk Tank sample will not have any bearing upon the result.

BMT antibody results can be used to assess BVDV risk and guide control programs, further, once a herd's baseline antibody level has been established, it can provide a highly sensitive method of surveilling for biosecurity failures. We are still working out Pestigard's impact on BMT antibody levels. Luckily for surveillance, Pestigard does not appear to have a substantial impact on BMT antibody levels.

- **Interpreting S/P Ratios**
  - **S/P > 1.0**
    - indicates active or recent PI exposure
  - **S/P > 0.75**
    - relatively recent herd BVDV exposure
  - **S/P > 0.25**
    - some random or historic BVDV exposure
  - **S/P < 0.25**
    - naïve herd

Swans Veterinary Services Pty Ltd ABN: 19 079 755 356 - PO Box 1514 Esperance WA 6450
Telephone: (08) 90 715 777 Fax (08) 90 715 057 Email: admin@swansvet.com

The initial S/P ratios can guide a producer's veterinarian at developing the most cost effective strategy for implementing a systematic BVDV control program utilizing appropriate testing, vaccination, biosecurity, and surveillance.

If a dairy has an S/P ratio over 1.0, then it is likely that either there is a lactating PI, or there was recently. To neutralize BVDV within the dairy:

1. Confirm that none of the lactating cows are PI.
   BMT PCR, ear notch testing, or rule out by testing calves.
2. Ear notch test all calves in the calf barns.
3. Confirm that no replacement heifers are PI, prior to mating and/or prior to coming into milk.

If a diary has an S/P ratio under 1.0 but above .75, then the likelihood of a lactating PI is low.

1. Ear notch test all calves in the calf barns. Test the mothers of any positive calves.
2. Confirm that no replacement heifers are PI, prior to mating and/or prior to coming into milk.

If a dairy has an S/P ratio below .75 but over .25, then there has been some past BVDV exposure.

1. Confirm that no replacement heifers are PI, prior to mating and/or prior to coming into milk.

If a dairy has an S/P ratio below .25, they are likely BVDV free.

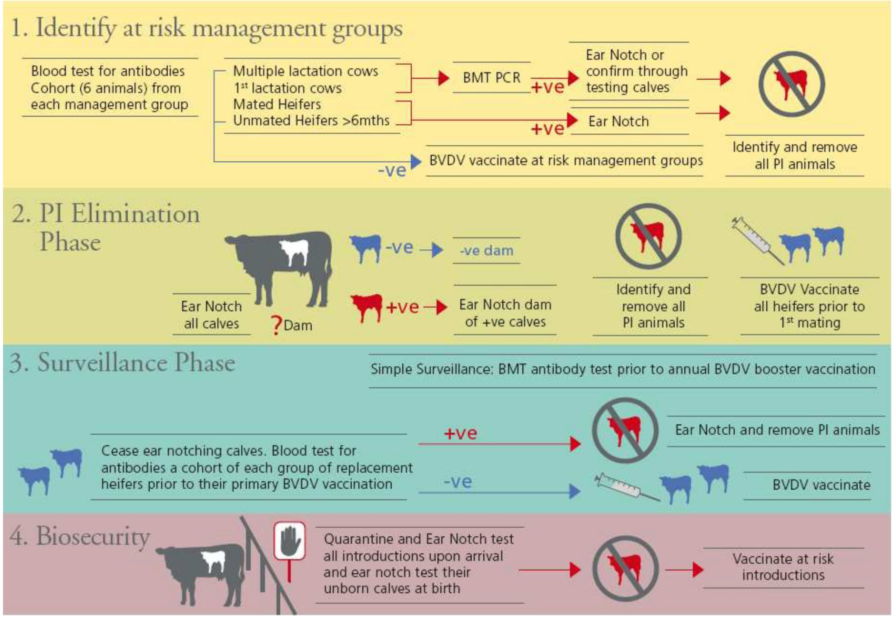

BMT testing fits quite well into our Dairy eradication control program. Once the S/P ratio of a dairy has been established, we can then focus on reducing that S/P ratio over time due to our interventions. Further by establishing an appropriately timed surveillance strategy we can monitor for biosecurity breaches. If an individual dairy's S/P ratio increases significantly, either a PI has been exposed to the dairy or a group of highly seropositive animals have entered the milking string, either way it is worth investigating.

Thank you for choosing to work with Swans Veterinary Services. We will continue to strive to offer the most complete BVDV consultancy and laboratory testing service possible.

Faithfully Yours,

The Swans Veterinary Services Team
